# Percutaneous Nephrolithotripsy in Morbidly Obese Patient: A Case Report

**DOI:** 10.1155/2022/5899896

**Published:** 2022-12-31

**Authors:** Fadhel Yusuf, Amr Elmekresh, Senthil Kumar, Fariborz Bagheri

**Affiliations:** Department of Urology, Dubai Hospital, Dubai, UAE

## Abstract

A 50-year old male patient with morbid obesity was admitted for removal of large staghorn calculi and multiple small stones in the left kidney. The patient was managed by Percutaneous Nephrolithotomy (PCNL). Surgery was carried out in prone position and Alken's metal dilators were used for tract dilation. Alken dilators were inserted without any challenges, and the procedure was completed in a shorter span of time than anticipated with total operative time of 2 hours, including the change of positioning from lithotomy to prone. No intra-operative or post-operative complications were encountered. The patient has been followed up for 6 months post-operatively, without any complications or any evidence of stone recurrence.

## 1. Introduction

The incidence of obesity has been dramatically increasing globally, in both developed and developing countries [1]. Consequently, there has been a significant rise in the number of obesity-related conditions, where obesity is the primary cause or is a vital contributing factor.

Obesity has been linked to the occurrence of several comorbidities such as diabetes mellitus, hypertension, and atherosclerosis. Insulin resistance, which is a significant consequence of obesity, constitutes one of the most important risk factors for renal stone formation [2].

Extracorporeal Shock Wave Lithotripsy (ESWL), Percutaneous Nephrolithotomy (PCNL), and Ureteroscopy (URS) are the most frequent current treatments for renal stones [3]. However, in obese patients, the use of ESWL is restricted due to the skin to stone distance. Alternatively, RIRS is a safe and effective method for treating renal stones in obese people, although it takes a long time to perform and multiple sessions is needed depending on the size, quantity, and location of the stones.

For renal stones greater than 2 cm, PCNL is the gold standard first-line treatment, particularly for obese people [3]. It is a minimally invasive technique which is successfully being used for the management of multiple calculi, staghorn calculi, or large calculi in lower calyx [4, 5].

Management of renal stones in morbidly obese patients can be challenging for the surgeon and anesthesia team. PCNL is a particularly important therapeutic modality for obese individuals and has revealed similar outcomes for obese and nonobese patients, although some technical modifications are suggested for treating morbidly obese patients [6]. This article is a case report of a morbidly obese patient presenting with a large kidney stone which was managed by PCNL using Alken technique for tract dilatation.

## 2. Case Presentation

A 50 years old Russian gentleman was referred to our facility from another hospital due to large left renal stone seen on ultrasound study, following active complaint of left flank pain for more than 1 year. He was a known case of recurrent kidney stones, and type II diabetes mellitus (DM). Additionally, he acquires a morbidly obese body habitus, with weight of 185 kg, height of 172 cm, and a BMI of 62 kg/m^2^. To assess further risk factors, when asked about his social habits and lifestyle, he stated has lives a normal active lifestyle, with a career in company management. Additionally, he stated that he tries to drink about 3 liters of water on a daily basis. His creatinine level and glomerular filtration rate (GFR) were within normal range during his multiple office visits.

**Figure 1:**
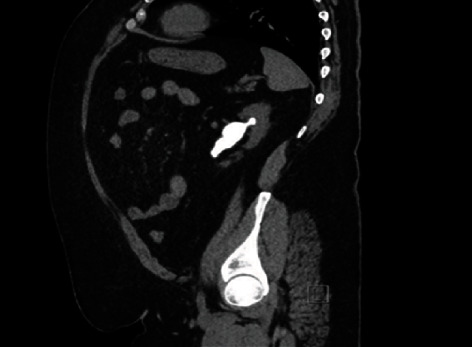
CT KUB demonstrating left staghorn kidney stone.

**Figure 2:**
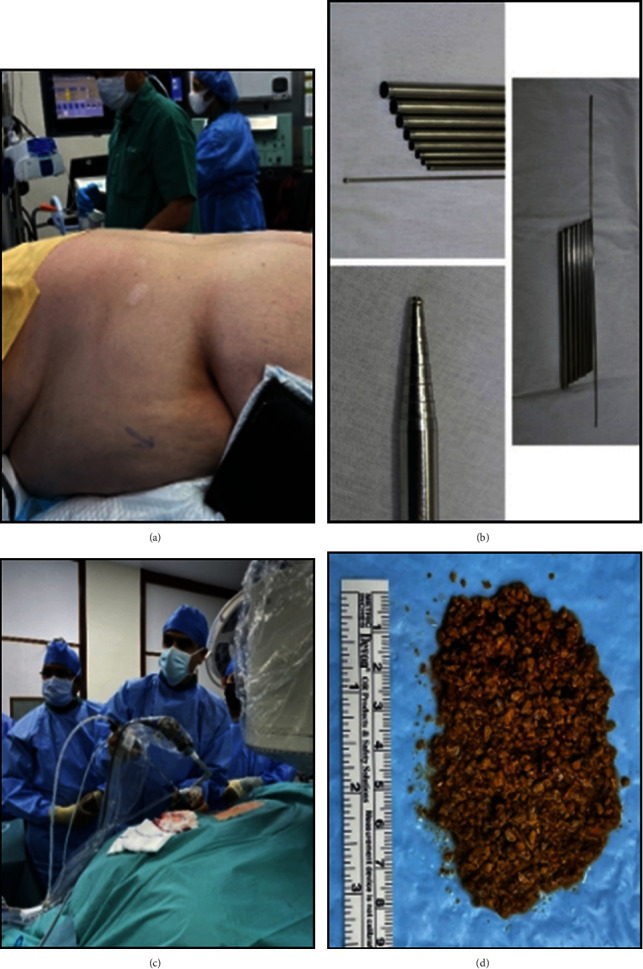
(a) Patient in prone positioning. (b) Alken dilators. (c) PCNL procedure. (d) Patient's stone fragments.

CT abdomen and pelvis with contrast was carried which showed small left kidney with staghorn stone of about 7 cm in diameter and multiple smaller stone in the calyces (Figure 1). Right kidney and ureter were free of stones. He was then offered to undergo PCNL, as the ultimate therapy for his renal stone (Figure 2).

A major challenge faced intraoperatively, was safe positioning of the patient due to his body habitus, which required additional care and time. Intraoperatively, in lithotomy position, left retrograde pyelography was done which showed large staghorn stone in the left renal pelvis and the calyces. Ureteral catheter was inserted and fixed externally. This was connected to normal saline and 1% methylene blue.

He was shifted to prone position. Under fluoroscopy guidance, the lower calyx was punctured, and a guide wire (Sensor from Boston Scientific) was inserted down to the left lower ureter.

A 10/8 French Teflon dilator was inserted down to the renal pelvis. Accordingly, a stiff guide wire could be placed next to the sensor guide wire which was used for dilatation of the tract by the Alken technique. The sensor guide wire was kept as a safety wire. Finally, the nephroscope was inserted through the stiff guide wire. All the stones were easily fragmented and aspirated by ultrasound probe of lithoclast machine (from EMS). Nephroscopy and fluoroscopy did not reveal any residual stones. There was no any difficulty in stone fragmentation and clearance comparing to other PCNL cases in prone position. Additionally, there was no need for extra long nephroscope or flexible nephoscope. An 18 Fr. Malecot nephrostomy tube was inserted. The total operative time was about 2 hours (Total operative time counts from the moment when cystoscope was inserted into the bladder in lithotomy position, continues with shifting the patient to prone position, and finishing with closure of skin incision) (Figure 2). Similar padding was used for this procedure as any other prone PCNL procedures and there was no need for any extra padding. The whole procedure was performed by an endourologist expert with 25 years of experience in urology and more than 500 cases of prone PCNL procedures.

Intra- and postoperative periods were uneventful.

He remained hospitalized for a total of four days, nephrostomy tube was clamped on day three, and as he was afebrile and pain-free, it was removed prior to discharge. Throughout the admission, creatinine followed a normal trend. Furthermore, his kidney stone analysis revealed uric acid stone composition.

He was further followed up after 2 weeks postoperatively. Bedside ultrasonography was done and showed unremarkable left kidney with no hydronephrosis or stones.

## 3. Discussion

In the recent decades, a significant rise in obesity has been observed worldwide. An increase in BMI has been positively associated with nephrolithiasis as well, with varying prevalence ranging from 3-20% reported across the literature, with majority of the stones identified as oxalate and uric acid stones [7–9]. In a study conducted by Taylor and Curhan which investigated the corelation of BMI on stone composition, observing for urinary excretion of various stone compositions, participants with higher BMI had a positive association with excretion of both oxalate and uric acid [10]. This complements uric acid stone analysis finding on our patient. The exact reason behind this association is multifactorial, however, the most hypothesized causes are failure of following a balanced dietary habit which leads to excess consumption of lithogenic food, and insulin resistance, as was the case in our patient who had a background of type II diabetes as well [11].

The most common surgical procedures currently in practice are ESWL, PCNL, and URS [3]. Amongst the aforementioned procedures, ESWL is the least-invasive procedure, which is more popular amongst eligible patients. However, this procedure carries a low feasibility amongst obese and morbidly obese patients. Several studies have reported on high rates of failure of stone removal with ESWL technique, particularly with increased skin-to-stone distance (SSD), leaving residual stone fragments behind [12–14].

RIRS is a preferable alternative option for obese and morbidly obese patient as there are no contraindications for, with no safety concerns [15]. However, in our case, the patient had a renal stone size of about 7 cm which is not an indication for RIRS, and is a candidate for PCNL [3].

The application of PCNL in obese patients may be challenging for surgeons. Obese patients present several technical challenges including anesthesia, patient positioning, imaging for access, longer skin-to-collecting-system distances, and nephrostomy tube dislodgement [16]. Traditionally, prone position is preferred for PCNL as it allows a direct access to the posterior calyx, and provides a safer procedure regarding the location of the bowel [17, 18]. Although a supine approach is suggested for obese patients due to a risk of ventilatory compromise in them, this position increases the working distance making it particularly difficult to rupture the upper pole. It also decreases the filling of the collecting system, making it constantly collapsed, and thus nephroscopy tends to be more difficult [19–21]. Hence, the use of supine position is still debatable. In the present case, PCNL was carried out in prone position and did not lead to any untoward respiratory events. Although most studies have noted similar outcomes in terms of stone-free rate, complications, and length of stay in obese versus nonobese patients, the only exception found was longer operative time in obese patients [6, 16]. Thus, minimizing surgical time in these patients may help prevent any cardiopulmonary complications that are anticipated to arise.

Dilatation of the percutaneous access tract is a critical step in PCNL procedure as it can lead to postoperative complications including bleeding and sepsis [20]. Dilation can be achieved by three standard techniques: semirigid (plastic or elastic) Amplatz dilatation, metal telescopic dilatation of the Alken type, and balloon dilatation. Balloon dilatation although has advantages of reduced complication rates and shorter durations of X-ray exposure, its application is limited due to high cost [19].

Plastic and metal dilators are inexpensive and have a higher chance of successful tract creation thus; these are widely used for tract dilation in PCNL. Studies comparing the use of plastic versus metal dilators revealed similar efficacy for both Alken dilator and Amplatz dilators [19, 22], commonly noted complications with metal dilators were the presence of more postoperative fevers >38.5°C and a higher rate of urosepsis. Amongst those with plastic dilators were more pleural injuries and subsequent blood loss, most likely occurring on withdrawal for insertion of the next larger dilator, leading to loss of compression, leading to subsequent bloos loss. On the other hand, telescopic Alken dilators exert pressure continuously, hence blood loss may be lower [19].

Another reported difference is the shorter tract formation time using Alken dilation was noted as compared to the plastic ones [22]. In our case too, the procedure was completed in a total of about two hours even though the patient was morbidly obese. Surgeon expertise certainly is a determining factor for the length of procedure however, the shorter duration of procedure can also be attributed to the use of Alken's telescopic metal dilators which are easier to insert and sequentially fit over the central metal rod to allow step wise dilation. The most challenging part of the procedure was changing the patient's position from lithotomy to prone position, which was managed safely and properly with help of 4 muscular messengers.

Although, there is no definite distinction as to which could be the best dilation technique to use in obese patients and surgeons may select different methods depending on their familiarity or experience with certain techniques, the use of metal dilators in the present case revealed positive results with no evidence of postop complications and a long stone free rate as determined endoscopically.

## 4. Conclusion

Obesity is a major risk factor for nephrolithiasis. In this case report we present a case of 50-year-old male with large staghorn left kidney stone which was managed with PCNL using Alken metal dilators which may provide an edge over plastic dilators by means of their ease of insertion and probable reduction in surgical time for patients undergoing PCNL which are critical, especially in obese patients.

## Supplementary Materials

Supplementary Materials The supplementary file uploaded is the CARE checklist for case reports. It delineates all the points needed for a case report, corresponding to the reported line as well.

## References

[1] Who (2009). *Obesity and overweight*.

[2] Kovesdy C. P., Furth S. L., Zoccali C., on behalf of the World Kidney Day Steering Committee (2017). Obesity and kidney disease: hidden consequences of the epidemic. *Journal of Renal Care*.

[3] Assimos D., Krambeck A., Miller N. L. (2016). Surgical management of stones: American urological association/endourological society guideline, part I. *Journal of Urology*.

[4] Preminger G. M., Assimos D. G., Lingeman J. E., Nakada S. Y., Pearle M. S., Wolf J. S. (2005). Chapter 1: AUA guideline on management of staghorn calculi: diagnosis and treatment recommendations. *The Journal of Urology*.

[5] Sabler I. M., Katafigiotis I., Gofrit O. N., Duvdevani M. (2018). Present indications and techniques of percutaneous nephrolithotomy: what the future holds?. *Asian Journal of Urology*.

[6] Zhou X., Sun X., Chen X. (2017). Effect of obesity on outcomes of percutaneous nephrolithotomy in renal stone management: a systematic review and meta-analysis. *Urologia Internationalis*.

[7] Negri A. L., Spivacow F. R., Del Valle E. E., Forrester M., Rosende G., Pinduli I. (2008). Role of overweight and obesity on the urinary excretion of promoters and inhibitors of stone formation in stone formers. *Urological Research*.

[8] Daudon M., Lacour B., Jungers P. (2006). Influence of body size on urinary stone composition in men and women. *Urological Research*.

[9] Curhan G. C., Willett W. C., Rimm E. B., Speizer F. E., Stampfer M. J. (1998). Body size and risk of kidney stones. *Journal of American Society of Nephrology*.

[10] Taylor E. N., Curhan G. C. (2006). Body size and 24-hour urine composition. *American Journal of Kidney Diseases*.

[11] Carbone A., Al Salhi Y., Tasca A., Palleschi G., Fuschi A., De Nunzio C. (2018). Obesity and kidney stone disease: a systematic review. *Minerva Urologica e Nefrologica*.

[12] Delakas D., Karyotis I., Daskalopoulos G., Lianos E., Mavromanolakis E. (2003). Independent predictors of failure of shockwave lithotripsy for ureteral stones employing a second-generation lithotripter. *Journal of Endourology*.

[13] Pareek G., Hedican S. P., Lee F. T., Nakada S. Y. (2005). Shock wave lithotripsy success determined by skin-to-stone distance on computed tomography. *Urology*.

[14] Perks A. E., Schuler T. D., Lee J. (2008). Stone attenuation and skin-to-stone distance on computed tomography predicts for stone fragmentation by shock wave lithotripsy. *Urology*.

[15] Laclergerie F., Jacquemet B., Guichard G. (2014). Flexible ureterorenoscopy in obese patients: results from a large monocenter cohort. *Progrès en Urologie*.

[16] Keheila M., Leavitt D., Galli R. (2016). Percutaneous nephrolithotomy in super obese patients (body mass index ≥ 50 kg/m2): overcoming the challenges. *BJU International*.

[17] Yuan D., Liu Y., Rao H. (2016). Supine versus prone position in percutaneous Nephrolithotomy for kidney calculi: a meta-analysis. *Journal of Endourology*.

[18] Zhang X., Xia L., Xu T., Wang X., Zhong S., Shen Z. (2014). Is the supine position superior to the prone position for percutaneous nephrolithotomy (PCNL)?. *Urolithiasis.*.

[19] Bryniarski P., Stelmach P., Taborowski P. (2016). Percutaneous nephrolithotomy with Amplatz and Alken dilators: an eight-year single tertiary care centre experience. *Medical Science Monitor*.

[20] Handa R. K., Matlaga B. R., Connors B. A. (2006). Acute effects of percutaneous tract dilation on renal function and structure. *Journal of Endourology*.

[21] Nour H. H., Kamal A. M., Ghobashi S. E., Zayed A. S., Rushdy M. M., El-Baz A. G. (2013). Percutaneous nephrolithotomy in the supine position: safety and outcomes in a single-centre experience. *Arab Journal of Urology*.

[22] Ozok H. U., Sagnak L., Senturk A. B., Karakoyunlu N., Topaloglu H., Ersoy H. (2012). A comparison of metal telescopic dilators and Amplatz dilators for nephrostomy tract dilation in percutaneous nephrolithotomy. *Journal of Endourology*.

